# Effectiveness of COVID-19 Vaccine Boosters in Children Across Pandemic and Endemic Periods

**DOI:** 10.3390/microorganisms14040883

**Published:** 2026-04-14

**Authors:** Eduardo A. Oliveira, Maria Christina L. Oliveira, Hercílio Martelli-Júnior, Fabrício Emanuel S. Oliveira, Daniella R. B. Martelli, Rayner Santos, Robert H. Mak, Ana Cristina Simões e Silva, Lilian M. Diniz, Cristiane S. Dias, Lays R. C. Foligno, Rafaela R. Herrerias, Ana Livia O. Andrade, Isabella O. Barbosa, Enrico A. Colosimo

**Affiliations:** 1 Health Sciences Postgraduate Program, Department of Pediatrics, School of Medicine, University Federal of Minas Gerais (UFMG), Belo Horizonte 30130-100, MG, Brazil; chrismariana@gmail.com (M.C.L.O.); acssilva@hotmail.com (A.C.S.e.S.); lilianmodiniz@gmail.com (L.M.D.); profacristianedias@gmail.com (C.S.D.); laysribeiroc@gmail.com (L.R.C.F.); rafaelarherrerias11@gmail.com (R.R.H.); analivia2003.andrade@gmail.com (A.L.O.A.); isabellaob30@gmail.com (I.O.B.); 2 Health Science/Primary Care Postgraduate Program, State University of Montes Claros (Unimontes), Montes Claros 39401-089, MG, Brazil; hmjunior2000@yahoo.com (H.M.-J.); fabricioemanuel1@hotmail.com (F.E.S.O.); daniellareismartelli@yahoo.com.br (D.R.B.M.); 3 Department of Statistics, University Federal of Minas Gerais (UFMG), Belo Horizonte 31270-901, MG, Brazil; rayner.santos10@gmail.com (R.S.); enricoc57@gmail.com (E.A.C.); 4 Department of Pediatrics, Division of Pediatric Nephrology, University of California, San Diego, CA 92110, USA; romak@health.ucsd.edu

**Keywords:** COVID-19, children, outcomes, vaccine, effectiveness

## Abstract

In the SARS-CoV-2 endemic phase, assessing the effectiveness of COVID-19 booster doses in children is essential for public health policy. This study evaluated the vaccine effectiveness (VE) of three doses (primary series plus booster) against severe outcomes, comparing the pandemic and endemic periods and children with and without comorbidities. We carried out a cohort study based on the population, utilizing comprehensive Brazilian data from individuals under 18 years of age with confirmed SARS-CoV-2 infection, spanning from February 2020 to June 2025. The primary exposure of interest was three or more doses of COVID-19 vaccines. The primary outcome of interest was COVID-19-related death. VE and the number needed to vaccinate (NNV) to prevent one death were estimated in a propensity score-matched cohort, with adjustments for confounders. Among 3,730,007 reported pediatric cases, 5472 (0.1%) died, 99% of whom did not receive a booster dose. During the pandemic, the VE against death was higher in children with comorbidities (92.7% [95% CI, 63.5–99.0]; NNV = 23 [19–30]) than in those without (68.2% [25.7–86.4]; NNV = 2000 [1111–9774]). During the endemic period, the VE against death remained high and was comparable between groups: 89.4% (29.8–98.7) and 75.8% (36.4–95.7) for children with and without comorbidities, respectively. Nevertheless, NNV levels were significantly lower in children with comorbidities, reflecting an increased risk at baseline. Although booster doses continue to offer substantial protection against fatal COVID-19 outcomes, the magnitude of this benefit is directly correlated with the baseline risk. Consequently, these findings support the implementation of risk-based prioritization strategies in public health decision-making for children.

## 1. Introduction

On 5 May 2023, the World Health Organization (WHO) declared the end of COVID-19 as a Public Health Emergency of International Concern [1]. SARS-CoV-2 has since transitioned to an endemic state, circulating similarly to other seasonal respiratory viruses [2,3,4]. The epidemiological pattern of COVID-19 has shifted as both the virus itself and immunity within the population have undergone changes [5]. Consequently, vaccination strategies have shifted from mass campaigns to periodic boosters, similar to the influenza vaccination schedules. However, the evolving immunological landscape of the endemic era remains uncertain [6,7,8], preventing a global consensus on the target populations for annual booster vaccinations, especially in children [9].

This uncertainty is reflected in the divergent national vaccination policies for pediatric populations. The US Food and Drug Administration (FDA) has established a framework focusing on adults over 65 years of age and individuals aged six months and older with risk factors [10]. In contrast, the American Academy of Pediatrics (AAP) recommends vaccination for high-risk children and all infants aged 6–23 months, while making it available to any child aged 2–18 years upon parental request [11]. Similarly, Brazil’s Ministry of Health includes COVID-19 vaccination for children aged 6 months to 5 years in its national program but reserves annual boosters for at-risk groups (https://www.gov.br/saude/pt-br/vacinacao, accessed on 19 November 2025). Despite this, children still face severe cases of COVID-19, with recent statistics showing that only a small fraction of hospitalized children in the United States were vaccinated [12].

Previous studies, conducted predominantly during the pandemic phase, established that pediatric COVID-19 vaccines have a favorable safety profile and provide moderate protection against infection and severe disease [13,14,15]. However, robust large-scale evidence from the post-pandemic era remains scarce, hindering informed public health policy in the endemic phase [16,17,18,19]. Recently, we investigated the vaccine effectiveness (VE) in children and adults with diabetes mellitus in the endemic period [20,21]. In this study, we expanded the scope by incorporating children with major comorbidities into the analysis. Therefore, we carried out a retrospective cohort study based on the population, which included more than three million Brazilian children and adolescents who had laboratory-confirmed SARS-CoV-2 infections. Our primary objective was to evaluate the VE against severe outcomes across both pandemic and endemic periods, specifically by comparing the VE in children with and without comorbidities.

## 2. Materials and Methods

### 2.1. Study Design, Participants, Data Sources, and Period

**Figure 1:**
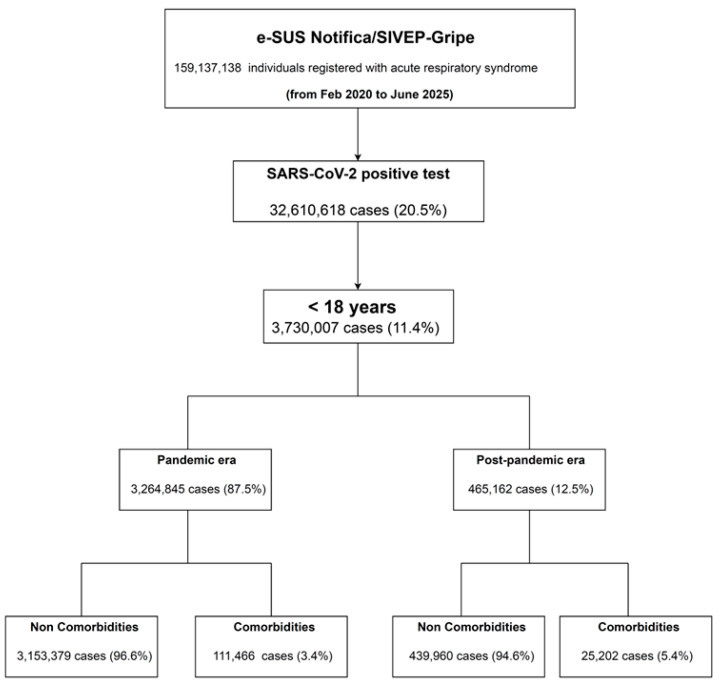
Flowchart of cohort selection.

Using data from two official Brazilian national COVID-19 surveillance systems provided by the Ministry of Health, we carried out a retrospective cohort study based on population data: (1) e-SUS Notifica, which tracks non-hospitalized individuals, and (2) SIVEP-Gripe, which monitors hospitalized individuals. Our investigation included every person aged < 18 years with laboratory-confirmed SARS-CoV-2 infections documented in these systems between 20 February 2020 and 30 June 2025. To be included in the systems, all cases had a confirmed diagnosis of COVID-19 via positive quantitative reverse transcription-polymerase chain reaction (RT-qPCR) test results or antigen test results. All information on both systems is available at https://dadosabertos.saude.gov.br/dataset?tags=covid-19, accessed on 3 June 2025). The selection process for cases included in our analysis from these comprehensive data sources is shown in a flowchart (Figure 1).

### 2.2. Covariates

We gathered demographic information, including age, sex, ethnicity, and geographic location. Age was divided into three categories: 0–4 years, 5–11 years, and 12–17 years. Ethnicity was classified into five categories based on the Brazilian Institute of Geography and Statistics (IBGE) classification: White, Black, Pardo, Asian, and Indigenous [22]. The geographic areas were divided into five official Brazilian macro-regions (North, Northeast, Central West, Southeast, and South), each with unique historical, socioeconomic, and healthcare system characteristics [23].

The clinical information gathered comprised the date when COVID-19 symptoms began, the date of hospital admission, the initial signs and symptoms presented, and the existence of comorbidities. We identified comorbidities using specific database fields for pre-existing chronic conditions, including diabetes, asthma, obesity, immunodeficiency, and cardiac, pulmonary, renal, neurological, and oncohematological diseases. For analysis, we classified comorbidity status as a dichotomous variable (present/absent) and a categorical variable (none, one, and two or more).

The study timeframe was empirically divided into two distinct periods: the pandemic period (1 February 2020 to 31 December 2022) and the endemic period (1 January 2023 to 30 June 2025). This division aligns with the reduction in the number of reported cases and COVID-19-related fatalities in Brazil. For each instance, the date of symptom onset was used as a reference point to determine the period. If this information was unavailable, the date of the SARS-CoV-2 test was used as the reference date.

### 2.3. Vaccination Status

Following the emergency use authorization issued by the Brazilian Health Regulatory Agency (ANVISA) for mRNA (Pfizer–BioNTech) and inactivated virus (CoronaVac) vaccines, a national vaccination campaign was initiated in a stepwise manner for the pediatric population, beginning with adolescents on 2 September 2021. Further details of the Brazilian vaccination program are available in the Supplementary Material (S.1).

Vaccination status was assessed by counting the number of vaccine doses administered at least 14 days prior to the appearance of SARS-CoV-2 infection symptoms, which is the necessary time frame for the immune system to respond. Individuals were categorized into four groups: (1) not vaccinated, (2) received one dose, (3) received two doses, and (4) received three or more doses of vaccine. In our cohort of 3,730,007 individuals, 2,759,411 were unvaccinated (74.0%), 179,001 had received one dose (4.8%), 421,044 had received two doses (11.3%), and 77,974 had received three or more doses (2.1%); for 235,396 individuals (6.0%), the vaccination status was unknown. Among the 683,157 vaccinated individuals, 455,690 received Pfizer–BioNTech (66.7%), 120,554 received CoronaVac (17.6), and 457 adolescents received viral vector vaccines (ChAdOx1 nCoV-19, Astrazeneca, Oxford, UK) not authorized for children. The booster dose was almost exclusively with Pfizer–BioNTech (97.6%). Details regarding the management of misclassifications in vaccination status and missing vaccination dates are provided in the Supplementary Material (S.2). A sensitivity analysis was also conducted to address issues related to missing vaccination dates, as detailed in Section S.2.

### 2.4. Primary Exposure

The main focus of interest was the status of being fully vaccinated, which included the initial series of two shots along with any booster doses.

### 2.5. Outcomes

The primary outcome was COVID-19-related mortality. We also assessed VE for the following secondary outcomes: (i) hospitalization resulting from SARS-CoV-2 infection and (ii) severe COVID-19, which is a composite outcome defined by ICU admission, requirement for mechanical ventilation, or death. VE was calculated separately for two distinct periods (pandemic and endemic eras) and further categorized based on the presence of comorbidities, as determined from the baseline data in the notification system.

### 2.6. Statistical Analysis

Continuous variables were represented as medians with interquartile ranges (IQRs) or means with standard deviations, depending on suitability. Categorical variables were characterized by frequency and proportion. To compare groups, the chi-square test was employed for proportions, and the Mann–Whitney U test was used for medians.

Missing data. Complete data were not available for all covariates, namely, sex (0.1%), ethnicity (20%), comorbidities (8%), and vaccination status (6%). Patients with unknown vaccination status were excluded from the primary analysis. For comorbidities, given the nature of the national surveillance databases, in which the absence of a recorded condition is likely to indicate a true absence, we handled missing data in the comorbidity fields by imputing absence. This conservative approach was consistent with those of previous studies that used similar data sources [22,23]. We did not impute the data for sex and ethnicity. The number of available covariates for analysis is presented in Table 1.

Vaccine effectiveness (VE) against the outcomes of interest was estimated using multivariable logistic regression models. We developed separate models for the entire cohort and key subgroups stratified by comorbidity status (with and without) and time period (pandemic vs. endemic). In these models, the outcome was the dependent variable and vaccination status (unvaccinated, one dose, two doses, three or more doses) was the independent variable, with unvaccinated individuals as the reference group. All models were adjusted for age, sex, ethnicity, geographic macro-region, predominant viral strain, and year of diagnosis or admission.

To enhance this analysis and offer a reliable estimate of the most comprehensive vaccination schedule, we utilized propensity score matching (PSM), concentrating on individuals who had received three or more doses compared to those who were unvaccinated [24,25]. We conducted four distinct PSM analyses for the subgroups of interest (patients with and without comorbidities during the pandemic and endemic periods). In each PSM, the vaccination status (three or more doses versus no vaccination) served as the exposure variable. The groups were matched based on age, sex, ethnicity, geographic location, viral lineage predominance, and year of admission using 1:1 nearest-neighbor matching without replacement and a caliper width of 0.2 standard deviations [26,27]. After matching the estimated propensity scores, we evaluated the balance between the vaccinated and unvaccinated groups using standardized mean differences (SMDs) and graphical density plots of the propensity scores (Supplementary Materials (S.3)) [28]. To conduct a more thorough analysis that accounted for any remaining imbalances, we applied conditional logistic regression to the matched datasets. In this model, the outcome was the dependent variable, with vaccination status as the primary predictor, adjusted for potential residual confounding factors. Following propensity score matching (PSM), we calculated the average treatment effect (ATE) on the population from the matched dataset and determined the number needed to vaccinate (NNV) to prevent one outcome of interest [26,29]. Vaccine effectiveness (VE) was calculated for all analyses as (1 − adjusted odds ratio) × 100%, with results presented alongside the 95% confidence intervals (CI). Analyses were performed using the statistical software packages R (version 4.3.0), SPSS (version 29), and STATA (version 18). Statistical significance was set at *p* < 0.05.

### 2.7. Ethical Considerations

This study used already de-identified and publicly available data from the SIVEP-Gripe and e-SUS Notifica databases. The study protocol was approved by the Research Ethics Committee of UFMG (Federal University of Minas Gerais). Approval Number: 6.127.414.

## 3. Results

### 3.1. Epidemiological Overview

**Figure 2:**
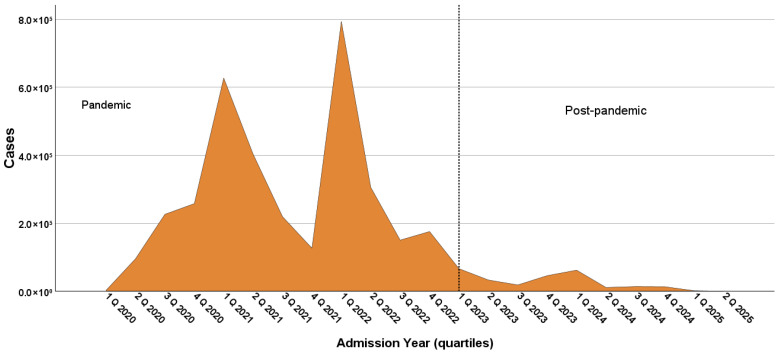
Temporal distribution of COVID-19 incident cases in children and adolescents from February 2020 to June 2025 in Brazil.

The epidemic curve in Figure 2 illustrates the total of 3,730,007 laboratory-confirmed pediatric COVID-19 cases registered from February 2020 to June 2025, highlighting that the majority of cases (3,264,845; 87.5%) were registered during the pandemic era, with a weekly average of 23,847 cases. In the endemic era, 465,162 (12.5%) cases were registered, corresponding to a weekly average of 3578 cases.

### 3.2. Clinical Characteristics and Outcomes

Table 1 presents the demographic and clinical features of the cohort stratified by the period of admission. The majority of cases (3,656,174; 98.0%) were reported via the eSUS Notifica system, with the remainder (73,833; 2.0%) from the SIVEP-Gripe database.

Of the 3,730,007 pediatric patients, 136,668 (3.7%) had at least one comorbidity, showing a modestly higher prevalence during the pandemic than during the endemic period. The endemic subgroup was younger, included a higher proportion from the Southeast region, and, consistent with the vaccine rollout, had higher rates of completed primary series or booster vaccinations (Table 1).

### 3.3. Vaccine Effectiveness (Entire Period)

**Figure 3:**
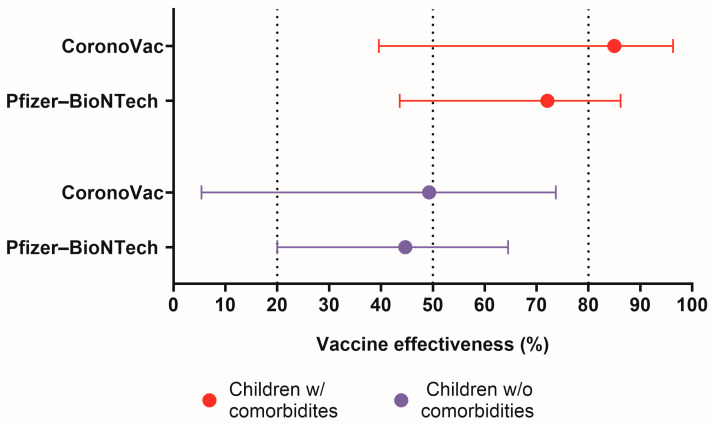
Comparative effectiveness of vaccine platforms stratified by children’s comorbidity status.

Over the entire study period, fully vaccinated individuals (defined as those who received three or more doses) had an adjusted vaccine effectiveness (VE) of 55.9% (95% CI, 52.3–59.3) against hospitalization, 53.1% (95% CI, 46.3–59.1) against severe COVID-19, and 54.2% (95% CI, 38.4–66.0) against death. Considering the vaccine platform and comorbidity status, the estimated 3-doses VE against death was similar between Pfizer and BioNTech and CoronoVac, with overlapping confidence intervals. For children with comorbities, the VE of CoronoVac was 85.0% (95% CI. 39.6–96.3) and of Pfizer–BioNTech was 72.1% (95% CI. 43.6–86.2). For children without comorbidities, the VE of CoronaVac was 49.3% (95% CI. 5.4–73.7) and that of Pfizer–BioNTech was 45.7% (95% CI 20.0–64.5) (Figure 3).

### 3.4. Vaccine Effectiveness (Pandemic vs. Endemic Eras)

**Figure 4:**
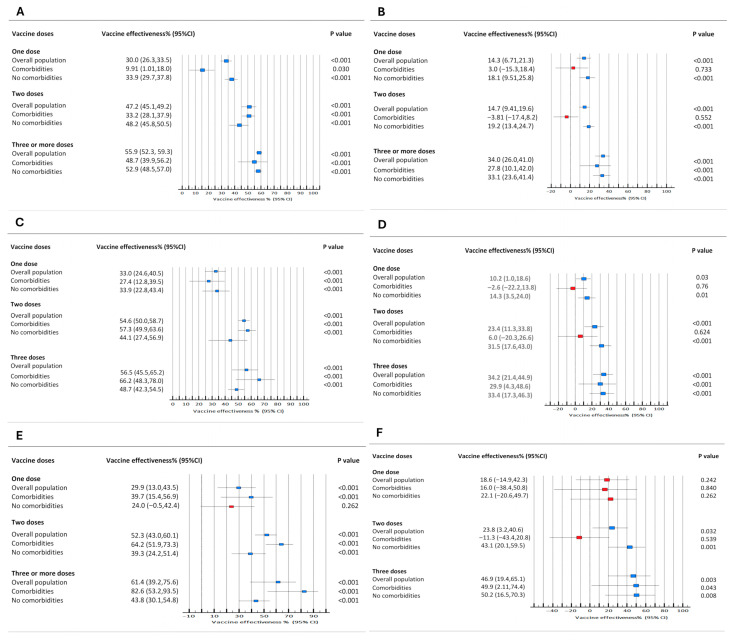
Vaccine effectiveness against outcomes of interest, stratified by study period. Outcomes are hospitalization (**A**,**B**), severe COVID-19 (**C**,**D**), and death (**E**,**F**). (**A**,**C**,**E**) represent the pandemic era; (**B**,**D**,**F**) represent the endemic era. All models were adjusted for age, sex, ethnicity, macro-region, predominant viral lineage, and year of admission (red square, *p* > 0.05, blue square, *p* < 0.05).

We compared vaccine effectiveness (VE) against the outcomes of interest during the pandemic and endemic periods. Figure 4 presents the results of 18 logistic regression models estimating VE against hospitalization (Panels A/B), severe COVID-19 (Panels C/D), and death (Panels E/F) for each period.

During the pandemic, most vaccination schedules provided significant dose-dependent protection against all outcomes. An exception was that a single dose did not confer significant protection against death in patients without comorbidities (Figure 4E). The highest VE was observed for the 3-dose schedule against death in the comorbidity cohort (82.6%; 95% CI: 53.2–93.5) (Figure 4E).

In the endemic era, the VE against all outcomes was lower for all vaccination schedules than during the pandemic. Notably, only schedules comprising three or more doses provided significant, albeit moderate, protection against all outcomes (Figure 4B,D,F). Importantly, for children without comorbidities, this booster schedule provided protection similar to that observed for children with comorbidities. For example, the VE against death was 49.9% (95% CI: 2.1–74.4) in children with comorbidities and 50.2% (95% CI: 16.5–70.3) in those without (Figure 4F).

We further assessed the effectiveness of booster doses using propensity score matching (PSM). After matching, we compared vaccine effectiveness (VE) against the outcomes of interest and the number needed to vaccinate (NNV) during the pandemic and endemic eras, stratified by the presence of comorbidities.

As shown in Table 2, the VE during the pandemic was consistently higher in children with comorbidities, exceeding 85% for all the outcomes. In contrast, the VE in children without comorbidities ranged from 53.4% for hospital admission to 68.2% for death. For example, against death, the VE was 92.7 (95% CI, 63.5–99.0) for children with comorbidities compared to 68.2% (95% CI: 25.7–86.4) for those without. This higher effectiveness in the high-risk group was reflected in a more favorable NNV. The NNV to prevent one death was 23 (95% CI, 19–30) for children with comorbidities versus 2000 (95% CI: 1114–9774) for those without comorbidities.

In the endemic era, VE remained statistically significant and similar between the cohorts, with overlapping confidence intervals (Table 2). For example, the VE against death was 89.4% (95% CI, 29.8–98.7) for children with comorbidities and 75.8% (95% CI, 36.4–95.7) for those without comorbidities. Despite the similar VE in the endemic era, NNV to prevent one death remained consistently lower in children with comorbidities (138 [95% CI, 73.0–1098] vs. 1297 [95% CI, 816–3162]).

## 4. Discussion

In a large-scale Brazilian cohort of 3.7 million pediatric patients, we evaluated the effectiveness of a vaccine booster dose (VE) against severe outcomes. The full vaccination schedule provided significant protection to the overall population after adjusting for confounders. We further stratified VE according to comorbidity status and across different periods of SARS-CoV-2 spread (pandemic versus endemic). As expected, the highest VE was observed with booster schedules (≥three doses) in individuals with comorbidities. Importantly, our findings underscore that booster schedules were significantly effective against all severity outcomes in both subgroups with and without comorbidities. A key finding in the current epidemiological landscape is that, in the endemic era, VE against severe outcomes was similar in all children, regardless of comorbidity status. However, although the VE against death was similar for children with (89.4% [95% CI, 29.8–98.7]) and without (75.8% [95% CI, 36.4–95.7]) comorbidities, the NNV was lower for those with comorbidities, reflecting their higher baseline risk.

Clinical trials have demonstrated that mRNA vaccines exhibit an excellent safety profile and high efficacy against COVID-19 in the pediatric population [30,31,32], leading to global implementation of pediatric vaccination campaigns. Subsequent real-world evidence has consistently demonstrated moderate effectiveness against SARS-CoV-2 infection and moderate protection against severe COVID-19 [33,34,35,36,37,38]. Our analysis corroborates these findings, confirming significant protection against hospitalization and severe outcomes, particularly in children with comorbidities.

As the pandemic has transitioned to an endemic phase, estimating the VE against severe outcomes remains crucial for public health policy. Our study provides a comprehensive analysis in this new context, showing that mRNA booster doses continue to provide significant protection against severe disease. To the best of our knowledge, only one other study has assessed VE during a comparable post-pandemic period. This study showed that the updated BNT162b2 XBB vaccine was linked to a VE of 65% (95% CI, 36 to 81) in preventing hospitalizations or emergency/urgent care visits among children aged 5–17 years [16]. Our findings align with this observation; however, we observed a slower yet still significant vaccine effectiveness of approximately 55% against hospital admissions in pediatric recipients of the non-updated three-dose COVID-19 vaccine regimen.

From a public health perspective, our analysis found that the NNV to prevent one severe outcome in the endemic era was substantially lower in children with comorbidities than in those without comorbidities. This difference likely reflects the higher baseline risk of severe disease in this vulnerable population. As noted in the literature, the NNV is a context-specific and dynamic metric that is highly sensitive to background risk [39]. Furthermore, there are no defined thresholds for favorable NNV, and its generalizability is often limited [40]. Some researchers caution that because NNV does not capture indirect benefits, such as herd immunity, it may undervalue the full public health benefit of vaccination programs [41]. Therefore, the NNV should be interpreted cautiously and in conjunction with other measures when evaluating vaccine utility and cost-effectiveness. For instance, in our analysis, the NNV to prevent one hospital admission in the endemic era was favorable for all children, underscoring the broad benefits of booster doses across the entire pediatric population.

A key strength of this study is the use of Brazilian nationwide databases, which provide extensive and high-quality data on COVID-19 [36,37]. We increased the representativeness and statistical power of our study by incorporating a sizable population of hospitalized and non-hospitalized pediatric patients through the successful merging of two major national databases.

Our study has several limitations. (1) Generalizability and context dependency: Vaccine effectiveness (VE) is highly context-dependent and is influenced by the underlying population risk profile [36,42]. As this study was conducted in a middle-income country with significant social disparities, the generalizability of our findings to high-income countries may be limited. We have previously demonstrated that the in-hospital fatality rate among Brazilian pediatric patients is higher than that observed in high-income countries [23,43]. Consequently, evaluations of VE across populations with diverse socioeconomic backgrounds remain necessary. (2) Missing data on third and subsequent doses: Despite our best efforts, data on vaccination dates for the third or subsequent doses were unavailable in the eSUS-notifica database. To assess the potential impact of this gap, we conducted a sensitivity analysis comparing our primary findings (using the corrected vaccination status) with those of the original database, which had a misclassification rate of approximately 10%. This analysis showed that the impact on our VE estimates for three or more doses—our primary exposure of interest—was negligible, as misclassification predominantly affected individuals who received only one or two doses of the vaccine. (3) Absence of time since last dose: The lack of data on the time elapsed since the last vaccine dose remains a significant limitation. In an endemic context, waning immunity is a well-documented phenomenon that could introduce a downward bias in VE estimates, particularly if a substantial portion of the cohort was long past their primary series or last booster [44]. However, although immunity against infection declines rapidly, protection against serious outcomes (hospitalization and death) has been shown to be more stable, which minimizes the impact of this bias on our primary outcomes [45,46]. Nevertheless, the absence of interval-specific data limits our ability to distinguish between waning biological immunity and the inherent effectiveness of the vaccine in different pediatric subgroups. Because our analysis did not account for this interval, the reported effectiveness represents a time-averaged estimate that may not reflect the peak vaccine protection. This omission could introduce a downward bias in our overall effectiveness estimates if a large proportion of the cohort had experienced significantly diminished immunity by the time of the study. Nonetheless, the high VE against death observed during the endemic phase suggests that the magnitude of underestimation was likely small in this study. (4) Case ascertainment and underreporting: Although temporal and regional adjustments were incorporated into our models, variations in test availability across periods and regions may have influenced the case ascertainment and cohort composition. Studies have indicated that underreporting in children—particularly those aged 2 to 9 years—was significantly higher than in adults, partly because children often present with mild or asymptomatic cases that did not meet the testing prioritization criteria during periods of scarcity [47,48]. While the underreporting of mild cases may affect estimates of effectiveness against symptomatic infection, our study focused on estimating the VE against severe outcomes. In this context, the impact of underreporting is likely to be smaller because of the more comprehensive reporting of severe cases. (5) Confounding factors and administrative database constraints: Despite rigorous methodological efforts to control for potential confounding factors, including propensity score matching, residual and unmeasured confounding factors may persist. Our study relied on large-scale administrative healthcare datasets, which, while providing substantial sample sizes and broad population coverage, have inherent limitations [49,50]. These datasets often lack granular clinical data, such as disease severity markers, socioeconomic status, healthcare access, vaccination coverage variability, and urban/rural differences, all of them important factors for a more precise adjustment of baseline risk. Despite these constraints, the use of such datasets offers a robust real-world perspective on VE that would be logistically unfeasible using traditional prospective cohorts. (5) Omission of prior SARS-COV-2 infection status: Missing data also represents an inherent challenge in administrative databases. In our analysis, we were unable to include the prior SARS-CoV-2 infection status as a covariate due to missing data. In contrast to adults, immune protection against SARS-CoV-2 in children and adolescents with hybrid immunity remains poorly understood, and most studies have focused on protection against symptomatic infections [51]. Importantly, a robust systematic review and meta-regression demonstrated that individuals with hybrid immunity had the highest magnitude and durability of protection [52]. Although protection estimates waned within months against reinfection, they remained high and were sustained for hospital admission or severe disease, which were the targets of our study. Nonetheless, we recognize that this omission may bias the VE estimates to some extent, although the direction of bias is uncertain and the magnitude is likely to be relatively small [53].

## 5. Conclusions

Our analysis demonstrated that a complete COVID-19 vaccination series significantly reduced severe outcomes in the pediatric population after adjusting for confounding factors. During the pandemic phase, booster doses played a critical role in mitigating severe illness, particularly among children with comorbidities, while maintaining significant effectiveness in otherwise healthy children. In the current endemic period, booster-conferred protection remains highly relevant, with comparable benefits observed in both high-risk and healthy pediatric cohorts. However, from a public health perspective, the number needed to vaccinate (NNV) to prevent a single death was consistently lower among children with chronic conditions during the post-pandemic period. Consequently, our findings underscore the necessity of continued prioritization for high-risk groups to maximize the clinical and public health impact of vaccination strategies. For children without comorbidities, the benefits of booster doses may vary depending on baseline risks and the epidemiological context.

In the context of widespread vaccine hesitancy and politicization [54], there is a pressing need for robust real-world evidence to guide public health policy. As large-scale clinical trials are unlikely to be conducted in this endemic setting, our findings may provide critical data to contribute to this discussion. However, cautious interpretation is warranted, as unmeasured residual confounding, inherent in observational studies, may limit the scope of our conclusions. These results underscore the necessity of studies that include diverse populations and can inform health authorities in formulating effective vaccination strategies for children in the post-pandemic era.

## Figures and Tables

**Table 1:** Demographic and clinical characteristics of children and adolescents with laboratory-confirmed COVID-19 stratified according to the period of the admission (pandemic vs. post-pandemic).

Covariates *	Overall (%) (2020–2025) 3,730,007 (100.0)	Pandemic (%) (2020–2022) 3,264,845 (87.5)	Endemic (%) (2023–2025) 465,162 (12.5)	*p*-Value
Database (BD)				
eSUS-notifica	3,656,174 (98.0)	3,209,167 (98.3)	447,007 (96.1)	<0.001
SIVEP-Gripe	73,833 (2.0)	55,678 (1.7)	18,155 (3.9)	
Age (years)				
Mean (SD)	9.3 (5.2)	9.4 (5.0)	8.9 (5.8)	<0.001
Age group (years)				
0–4	815,101 (21.9)	678,532 (20.8)	136,583 (29.4)	<0.001
5–11	1,458,324 (39.1)	1,320,127 (40.4)	138,189 (29.7)	
12–17	1,456,582 (39.1)	1,266,186 (38.8)	190,390 (40.9)	
Sex (n = 3,724,913)				
Male	2,031,465 (54.5)	1,804,905 (55.4)	226,560 (48.7)	<0.001
Female	1,693,448 (45.5)	1,454,856 (44.6)	238,592 (51.3)	
Region				
Southeast	1,491,076 (40.0)	1,251,304 (38.3)	239,772 (51.5)	<0.001
South	932,709 (25.0)	865,324 (26.5)	67,385 (14.5)	
Central-West	377,031 (10.1)	312,753 (9.6)	64,278 (13.8)	
Northeast	636,856 (17.1)	575,516 (17.6)	61,340 (13.2)	
North	292,335 (7.8)	259,948 (8.0)	32,387 (7.0)	
Ethnicity (n = 2,965,946)				
White	1,648,737 (55.6)	1,458,309 (56.2)	190,428 (51.6)	<0.001
Brown	1,177,553 (39.7)	1,019,313 (39.3)	158,240 (42.8)	
Black	77,658 (2.6)	67,653 (2.6)	10,005 (2.7)	
Asian	61,288 (2.1)	50,744 (2.0)	10,544 (2.9)	
Indigenous	710 (0.0)	535 (0.0)	175 (0.0)	
Signs/symptoms at presentation				
Fever	1,520,927 (40.8)	1,291,646 (39.6)	229,281 (49.3)	<0.001
Cough	1,616,742 (43.3)	1,364,137 (41.8)	252,605 (54.3)	<0.001
Dyspnea	236,071 (6.3)	196,166 (6.0)	39,905 (8.6)	<0.001
Odynophagia	1,035,656 (27.8)	891,924 (27.3)	143,732 (30.9)	<0.001
Number of comorbidities				
None	3,593,339 (96.3)	3,153,379 (96.6)	439,960 (94.6)	<0.001
1	129,500 (3.5)	105,560 (3.2)	23,940 (5.1)	
2	6266 (0.2)	5173 (0.2)	1093 (0.2)	
3 or more	902 (0.0)	733 (0.0)	169 (0.0)	
Major comorbidities				
Pulmonary	58,735 (1.6)	50,384 (1.5)	8351 (1.8)	<0.001
Diabetes mellitus	7675 (0.2)	6860 (0.2)	815 (0.2)	<0.001
Obesity	5956 (0.2)	5367 (0.2)	589 (0.1)	<0.001
Cardiology	9405 (0.3)	7630 (0.2)	1775 (0.4)	<0.001
Asthma	6814 (0.2)	5131 (0.2)	1683 (0.4)	<0.001
Immunosuppression	6330 (0.2)	5201 (0.2)	1129 (0.2)	<0.001
Kidney	1557 (0.0)	1219 (0.0)	338 (0.1)	<0.001
SARS-CoV-2 strain				
Ancestral	609,332 (16.3)	609,332 (18.7)	0 (0.0)	<0.001
Gamma	1,087,021 (29.1)	1,087,021 (33.3)	0 (0.0)	
Delta	140,489 (3.8)	140,489 (4.3)	0 (0.0)	
Omicron	1,893,165 (50.8)	1,428,003 (43.7)	465,162 (100.0)	
Vaccine schedule (n = 3,494,611)				
None	2,759,411 (80.3)	2,579,212 (83.6)	180,199 (51.2)	<0.001
One	179,001 (5.2)	146,435 (4.7)	32,566 (9.2)	
Two	421,044 (12.2)	321,563 (10.4)	99,481 (28.3)	
Three or more	77,974 (2.3)	38,124 (1.2)	39,850 (11.3)	

* Data (n) in the first column represent the available data for those covariates with missing values (gender, ethnicity, and vaccine).

**Table 2:** Estimated vaccine effectiveness (VE) and number needed to vaccinate (NNV) for preventing one outcome.

Period	Outcomes/Groups	VE (%) (95% CI)	NNV (95% CI)
Pandemic	Hospitalization		
	Comorbidity cohort	87.5 (78.9–92.5)	5 (4–6)
	Non-comorbidity cohort	53.4 (43.8–61.4)	110 (93–135)
	Severe COVID-19		
	Comorbidity cohort	88.5 (75.0–94.7)	9 (7–11)
	Non-comorbidity cohort	58.7 (39.3–71.9)	465 (334–768)
	Death		
	Comorbidity cohort	92.7 (43.5–99.0)	23 (19–30)
	Non-comorbidity cohort	68.2 (25.7–86.4)	2000 (1114–9774)
Endemic	Hospitalization		
	Comorbidity cohort	55.1 (39.7–66.4)	22 (16–38)
	Non-comorbidity cohort	52.6 (43.6–60.6)	94 (80–113)
	Severe COVID-19		
	Comorbidity cohort	53.4 (28.9–69.5)	44 (28–987)
	Non-comorbidity cohort	35.8 (15.0–52.4)	385 (277–441)
	Death		
	Comorbidity cohort	89.4 (9.81–98.7)	138 (73–1098)
	Non-comorbidity cohort	75.8 (36.4–95.7)	1297 (816–3161)

VE, vaccine effectiveness; NNV (number needed to vaccinate). All *p*-values < 0.01.

## Data Availability

The data presented in this study are available in the Portal of Open Access do SUS (Ministério da Saúde do Brasil) at https://dadosabertos.saude.gov.br/ (accessed on 3 June 2025). These data were derived from the following resources available in the public domain: SIVEP-Gripe and e-SUS Notifica (https://dadosabertos.saude.gov.br/dataset?tags=covid-19), (accessed on 3 June 2025).

## Supplementary Materials

The following supporting information can be downloaded at: https://www.mdpi.com/article/10.3390/microorganisms14040883/s1, Figure S1: Sensitivity analysis comparing dataset with the corrected dataset. Estimated vaccine effectiveness against death stratified by comorbidity status and period: (A) pandemic era, original (non-adjusted) dataset; (B) pandemic era, adjusted dataset; (C) post-pandemic era, original (non-adjusted) dataset; and (D) post-pandemic era, adjusted dataset. All models were adjusted by age, sex, ethnicity, macro-regions, predominant viral lineage, and year of admission; Figure S2: Distribution of propensity scores for unvaccinated and vaccinated individuals in pandemic era. (A) patients with comorbidities, before matching; (B) patients with comorbidities, after matching; (C) patients without comorbidities, before matching; and (D) patients without comorbidities, after matching; Figure S3: Distribution of propensity scores for unvaccinated and vaccinated individuals in post-pandemic era. (A) patients with comorbidities, before matching; (B) patients with comorbidities, after matching; (C) patients without comorbidities, before matching; and (D) patients without comorbidities, after matching; Table S1: Vaccine authorizations and campaign rollout for the pediatric population in Brazil, 2021–2022; Table S2: Characteristics of vaccinated (3-doses) and controls (unvaccinated) before and after propensity matching; Table S3: Characteristics of vaccinated (3-doses) and controls (unvaccinated) before and after propensity matching; Table S4: Characteristics of vaccinated (3-doses) and controls (unvaccinated) before and after propensity matching; Table S5: Characteristics of vaccinated (3-doses) and controls (unvaccinated) before and after propensity matching.

## Abbreviations

The following abbreviations are used in this manuscript:

COVID-19	Coronavirus disease 2019
WHO	World Health Organization
SARS-CoV-2	Severe acute respiratory syndrome coronavirus 2
SIVEP-Gripe	Influenza Epidemiological Surveillance Information System
aOR	Adjusted odds ratio
CI	Confidence interval
NNV	Number needed to vaccinate
VE	Vaccine effectiveness
ICU	Intensive care unit
ATE	Average treatment effect
FDA	US Food and Drug Administration
IBGE	Brazilian Institute of Geography and Statistics
AAP	American Academy of Pediatrics
