# Supplementary material for: Effectiveness of COVID-19 Vaccine Boosters in Children Across Pandemic and Endemic Periods
Source: Microorganisms. 2026 Apr 14;14(4):883. doi: 10.3390/microorganisms14040883 (PMC13118569; doi:10.3390/microorganisms14040883)
Supplement: Supplementary file 1 [file microorganisms-14-00883-s001.zip › microorganisms-4224638-supplementary.pdf]

# Supplementary Material

*Effectiveness of COVID-19 Vaccine Boosters in Children Across Pandemic and Endemic Periods*

## Microorganisms

### Contents

**Supplementary Material (S.1)** **2**
**Supplementary Material (S.2)** **4**
**Supplementary Material (S.3)** **7**

**Supplementary Material (S.1)**

**Brazilian COVID-19 Vaccination Program for the Pediatric Population**

**1. Program Overview**

The Brazilian Ministry of Health (MS) was the sole provider of COVID-19 vaccines. Following emergency use authorization by the National Health Surveillance Agency (ANVISA), a national vaccination campaign was implemented sequentially for different age groups. The authorized vaccines for pediatric groups were BNT162b2 mRNA (Pfizer–BioNTech) and inactivated virus (CoronaVac) vaccines.

**2. Chronological Rollout for Pediatric Age Groups**

The following table summarizes the key dates for vaccine authorizations and initiation of vaccination campaigns for each pediatric age group in Brazil.

**Table S.1.** Vaccine authorizations and campaign rollout for the pediatric population in Brazil, 2021–2022.

| Age Group | Vaccine | Authorization Date | Campaign Start Date | Initial Schedule |
|---|---|---|---|---|
| **Adolescents (12-17 years)** | Pfizer-BioNTech | June 11, 2021 | September 02, 2021 | 2 doses |
| | CoronaVac | January 20, 2022 | January 20, 2022 | 2 doses |
| **Children (5-11 years)** | Pediatric Pfizer-BioNTech | December 16, 2021 | January 21, 2022 | 2 doses |
| | CoronaVac | January 20, 2022 | January 20, 2022 (from 6 y) | 2 doses |
| **Children (3-4 years)** | CoronaVac | July 13, 2022 | July 13, 2022 | 2 doses |
| **Children (6 m – 4y)** | Pediatric Pfizer-BioNTech | September 16, 2022 | September 16, 2022 | 3 doses |

***Subsequent Updates:***

- A booster (3rd) dose of Pfizer-BioNTech was made available for adolescents in May 2022 and for children (5-11 years) in January 2023.
- In January 2024, COVID-19 vaccines were formally incorporated into the National Immunization Program (PNI). For the pediatric population, this meant:
    - For children 6 months-4 years: Completion of schedules (Pfizer three doses or CoronaVac two doses).
    - For children 5-17 years: a single annual dose is recommended only for at-risk groups.

**3. Data Source and Variable Creation**

To ensure reliable data, the Ministry of Health updated the official databases in 2021, introducing a dedicated vacina_covid field with dates for each vaccine dose. From this field, we derived a four-category vaccination status variable for our analysis.

1. Unvaccinated
2. Partially vaccinated (1 dose)
3. Primary schedule completed (2 doses)
4. Boosted (3 or more doses)

A key limitation is that the exact vaccination dates were unavailable for individuals in the e-SUS Notifica database; the implications of this are addressed in our sensitivity analysis (Supplementary Material S.2)

## Supplementary Material (S.2)

*Sensitivity analysis*

During data cleaning, we identified and corrected a misclassification in the original dataset, where 61,581 individuals were erroneously listed as vaccinated, despite their recorded vaccination dates being after symptom onset. Most misclassifications were among individuals who received one dose(83%). Conversely, 97.5% of those who received three doses were correctly classified.

However, the exact vaccination dates for each dose were unavailable in the eSUS-notifica database for individuals who received three or more doses of vaccine. Therefore, while we used the recorded number of doses to assign individuals to this group, we could not verify the 14-day lag for each specific dose. To assess the potential impact of this data limitation and our corrections, we conducted a sensitivity analysis by comparing our corrected dataset with the original dataset

We conducted a sensitivity analysis using binary logistic regression models to examine the potential impact of vaccination status misclassification on vaccine effectiveness (VE) estimates. Figure S.1 shows the estimated VE against death in the original dataset and in the corrected dataset, across the periods of the study (pandemic and post-pandemic).

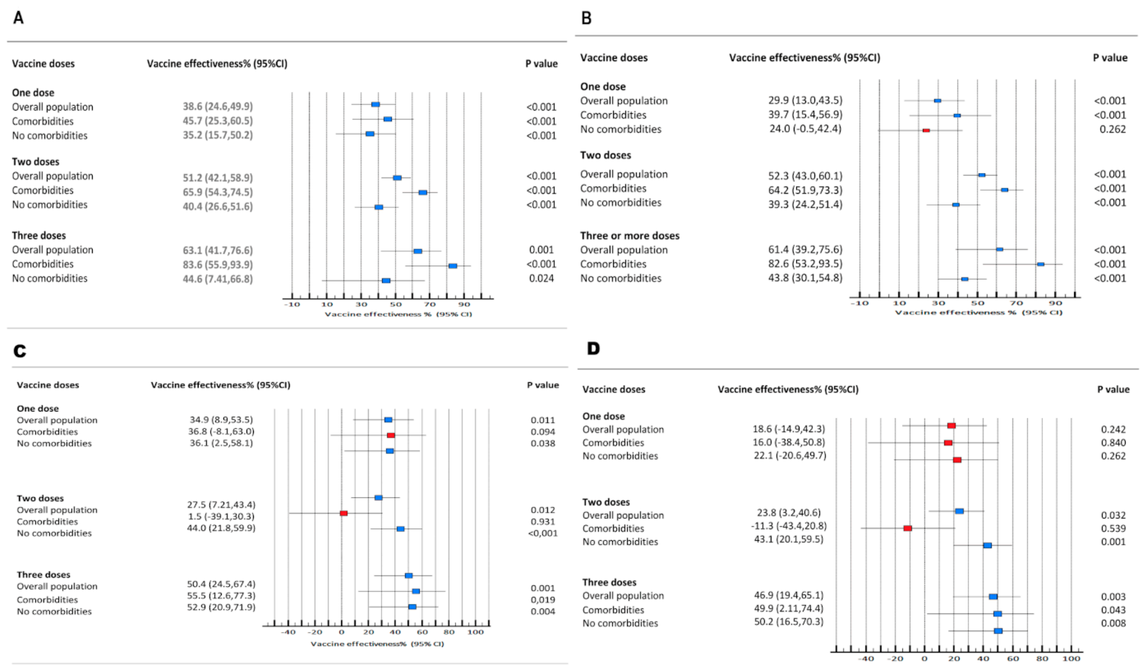


**Figure S1.** Sensitivity analysis comparing dataset with the corrected dataset. Estimated vaccine effectiveness against death stratified by comorbidity status and period: (A) pandemic era, original (non-adjusted) dataset; (B) pandemic era, adjusted dataset; (C) post-pandemic era, original (non-adjusted) dataset; and (D) post-pandemic era, adjusted dataset. All models were adjusted by age, sex, ethnicity, macro-regions, predominant viral lineage, and year of admission.

Compared to estimates from the corrected database, misclassification in the original data led to a systematic overestimation of vaccine effectiveness (VE), primarily among individuals who received one or two doses. For example, during the pandemic era among patients with comorbidities, VE against death for a 1-dose regimen was 45.7% (95% CI: 25.3 to 65.5) in the original dataset (Panel A) but 39.7% (95% CI: 15.4 to 56.9) in the adjusted dataset (Panel B). In contrast, VE estimates for three or more doses—our primary exposure of interest—remained highly consistent. For this group with comorbidities in the pandemic era, VE was nearly identical: 83.6% (95% CI: 55.9 to 93.9) in the original dataset versus 82.6% (95% CI: 53.2 to 93.5) in the adjusted dataset.

The same pattern was observed in the post-pandemic era. For patients with comorbidities who received a 1-dose regimen, VE against death was 36.8% (95% CI: -8.1 to 63.0) in the original dataset (Panel C) compared to 16% (95% CI: -38.4 to 50.4) in the corrected dataset (Panel D). Conversely, VE for three or more doses was similar in this era as well: 55.5% (95% CI: 12.6 to 77.6) in the original dataset versus 49.9% (95% CI: 2.1 to 74.4) in the adjusted dataset.

## Supplementary Material (S.3)

### *Propensity Score matching - Balance diagnosis*

In our observational study, we used propensity score matching (PSM) to reduce selection bias by balancing observed pre-intervention characteristics between the intervention group (3 doses of COVID-19 vaccine) and the control group (unvaccinated). We conducted four separate PSM analyses for the following subgroups:

(1) patients with comorbidities during the pandemic,

(2) patients without comorbidities during the pandemic,

(3) patients with comorbidities in the post-pandemic era, and

(4) patients without comorbidities in the post-pandemic era.

After matching on the estimated propensity score, we assessed balance between vaccinated and unvaccinated groups using standardized mean differences (SMD) and density graphical plots of propensity scores before and after matching.

Tables S2–S5 present the distributions of covariates for unvaccinated and vaccinated individuals in each subgroup, before and after matching. The matching procedure achieved excellent balance, as evidenced by post-matching standardized mean differences (SMDs) all close to zero and variance ratios (not shown) near 1.0 for all covariates. In the matched samples, the prevalences of baseline characteristics were highly similar between the treated and untreated groups. Consequently, selection bias due to observed confounders was substantially reduced, supporting the robustness of the subsequent treatment effect estimates.

Figure S2 displays the propensity score distributions for unvaccinated and vaccinated individuals, before and after matching, stratified by comorbidity status during the pandemic period. The near overlap of the densities indicates successful balancing of the propensity score distributions between the groups. Similarly, Figure S3 shows these distributions stratified by comorbidity status in the post-pandemic period. The results were comparable, with the propensity score densities showing near-complete overlap after matching.

**Table S2. Characteristics of vaccinated (3-doses) and controls (unvaccinated) before and after propensity matching.**

**Group: pandemic period, children with comorbidities**

| | **Before matching** | | | **After marching** | | |
|---|---|---|---|---|---|---|
| **Covariates*** | **Unvaccinated (%) 70,176 (97.4)** | **3-doses vaccine (%) 2,098 (2.6)** | **Standardized differences** | **Unvaccinated (%) 2,092 (50.0)** | **3-doses vaccine (%) 2,092 (50.0)** | **Standardized differences** |
| Age group (years) | | | | | | |
| 12 - 17 | 26,648 (33,7) | 1,327 (63,4) | reference | 1,327 (63,4) | 1,327 (63,4) | reference |
| 5 – 11 | 28,781 (36,4) | 97 (4,6) | -0.074712 | 97 (4,6) | 97 (4,6) | -0.0032434 |
| 0 - 4 | 23,747 (30,0) | 668 (31,9) | -0.004105 | 668 (31,9) | 668 (31,9) | 0.0020356 |
| Sex | | | | | | |
| Female | 36844 (46,5) | 1,205 (57,6) | reference | 1,205 (57,6) | 1,205 (57,6) | reference |
| Male | 42308 (53,5) | 887 (42,4) | 0.000890 | 887 (42,4) | 887 (42,4) | -0.001277 |
| Region | | | | | | |
| Southeast | 40,612 (51,3) | 1,291 (61,7) | reference | 1,291 (61,7) | 1,291 (61,7) | reference |
| South | 12,611 (15,9) | 246 (11,8) | 0.025194 | 246 (11,80 | 246 (11,80 | 0.001922 |
| Central-West | 7,033 (8,9) | 141 (6,7) | 0.031867 | 141 (6,7) | 141 (6,7) | -0.004916 |
| Northeast | 12,980 (16,4) | 358 (17,1) | -0.032493 | 358 (17,1) | 358 (17,1) | 0.002527 |
| North | 5,940 (7,5) | 56 (2,7) | 0.022690 | 56 (2,7) | 56 (2,7) | 0.003511 |
| Ethnicity | | | | | | |
| White | 32,553 (51,2) | 794 (54,5) | reference | 885 (51,80 | 794 (54,5) | reference |
| Brown | 27,286 (42,9) | 542 (37,2) | -0.088993 | 710 (41,5) | 542 (37,2) | 2.27e-16 |
| Black | 2,568 (4,0) | 74 (5,1) | 0.024028 | 78 (4,6) | 74 (5,1) | -0.005965 |
| Asian/ Indigenous | 1,152 (1,8) | 47 (3,2) | 0.069502 | 36 (2,1) | 47 (3,2) | -0.001994 |

**Table S3. Characteristics of vaccinated (3-doses) and controls (unvaccinated) before and after propensity matching.**

**Group: pandemic era, children without comorbidities :**

| | **Before matching** | | | **After marching** | | |
|---|---|---|---|---|---|---|
| **Covariates[*]** | **Unvaccinated (%)**<br>**2,500,036 (98.6)** | **3-doses vaccine (%)**<br>**36,032 (1.4)** | **Standardized differences** | **Unvaccinated (%)**<br>**36,032 (50.0)** | **3-doses vaccine (%)**<br>**36,032 (50.0)** | **Standardized differences** |
| Age group (years) | | | | | | |
| 12 - 17 | 798,,297 (31,9) | 27,037 (75,0) | reference | 27,037 (75,0) | 27,037 (75,0) | reference |
| 5 – 11 | 1,111,416 (44,5) | 1,601 (4,4) | -0.0355681 | 1,601 (4,4) | 1,601 (4,4) | 0.0005783 |
| 0 - 4 | 590,323 (23,6) | 7,394 (20,5) | -0.044653 | 7,394 (20,5) | 7,394 (20,5) | -0.0000932 |
| Sex | | | | | | |
| Female | 1,049,054 (42,0) | 21,142 (58,7) | reference | 21,142 (58,7) | 21,142 (58,7) | reference |
| Male | 1,448,449 (58,0) | 14,890 (41,3) | -0.0045451 | 14,890 (41,3) | 14,890 (41,3) | -0.0002237 |
| Region | | | | | | |
| Southeast | 869,020 (34,8) | 19,177 (53,2) | reference | 19,177 (53,2) | 19,177 (53,2) | reference |
| South | 751,849 (30,1) | 3,584 (9,9) | 0.0003711 | 3,584 (9,9) | 3,584 (9,9) | -9.20e-16 |
| Central-West | 222,384 (8,9) | 3,749 (10,4) | -0.0343242 | 3,749 (10,4) | 3,749 (10,4) | -0.0001245 |
| Northeast | 443,526 (17,7) | 8,305 (23,0) | 0.010963 | 8,305 (23,0) | 8,305 (23,0) | 0.000085 |
| North | 21,3257 (8,5) | 1,217 (3,4) | 0.0027462 | 1,217 (3,4) | 1,217 (3,4) | 0.0003779 |
| Ethnicity | | | | | | |
| White | 1,166,185 (57,4) | 794 (54,5) | reference | 12,804 (47,0) | 13,945 (51,1) | reference |
| Brown | 777,530 (38,3) | 542 (37,2) | -0.1455262 | 13,115 (48,1) | 11,165 (40,9) | 1.92e-15 |
| Black | 50,027 (2,5) | 74 (5,1) | 0.0180395 | 793 (2,9) | 879 (3,2) | 0.0006231 |
| Asian/Indigenous | 38,963 (1,9) | 47 (3,2) | 0.1547236 | 539 (2,0) | 1303 (4,8) | 1.66e-15 |

**Table S4. Characteristics of vaccinated (3-doses) and controls (unvaccinated) before and after propensity matching.**

**Group: post-pandemic era, children with comorbidities**

**:**

| | Before matching | | | After marching | | |
|---|---|---|---|---|---|---|
| **Covariates*** | **Unvaccinated (%) 10,631 (84.1)** | **3-doses vaccine (%) 2,009 (15.9)** | **Standardized differences** | **Unvaccinated (%) 1,866 (50.0)** | **3-doses vaccine (%) 1,866 (50.0)** | **Standardized differences** |
| Age group (years) | | | | | | |
| 12 - 17 | 1,526 (14,4) | 1,610 (80,1) | reference | 1,465 (78,5) | 1,467 (78,6) | reference |
| 5 – 11 | 2,786 (26,2) | 131 (6,5) | -0.032032 | 136 (7,3) | 131 (7,0) | 0.010801 |
| 0 - 4 | 6,319 (59,4) | 268 (13,3) | -0.035550 | 265 (14,2) | 268 (14,4) | -0.003890 |
| Sex | | | | | | |
| Female | 4,926 (46,3) | 1,124 (55,9) | reference | 1,009 (54,1) | 1,015 (54,4) | reference |
| Male | 5,704 (53,7) | 885 (44,1) | -0.036549 | 857 (45,9) | 851 (45,6) | -0.013775 |
| Region | | | | | | |
| Southeast | 5,394 (50,7) | 1,289 (64,2) | reference | 1,038 (55,6) | 1,182 (63,3) | reference |
| South | 1,537 (14,5) | 243 (12,1) | 0.019689 | 243 (13,0) | 237 (12,7) | 0.003905 |
| Central-West | 1,441 (13,6) | 157 (7,8) | -0.205876 | 262 (14,0) | 155 (8,3) | -2.02e-16 |
| Northeast | 1,522 (14,3) | 252 (12,5) | -0.053535 | 224 (12,0) | 224 (12,0) | 0.015051 |
| North | 737 (6,9) | 68 (3,4) | -0.082491 | 99 (5,3) | 68 (3,6) | 0.010233 |
| Ethnicity | | | | | | |
| White | 4,086 (48,0) | 901 (57,6) | reference | 681 (47,7) | 840 (57,6) | reference |
| Brown | 3,993 (46,9) | 517 (33,0) | -0.232171 | 634 (44,4) | 483 (33,1) | -0.007274 |
| Black | 287 (3,4) | 70 (4,50 | 0.016641 | 57 (4,0) | 63 (4,3) | 0.038757 |
| Asian/Indigenous | 139(1,7) | 77 (4,9) | 0.045975 | 57 (4.0) | 72 (4,9) | 0.039585 |

**Table S5. Characteristics of vaccinated (3-doses) and controls (unvaccinated) before and after propensity matching.**
**Group: post-pandemic era, children without comorbidities**
**:**

| | Before matching | | | After marching | | |
|---|---|---|---|---|---|---|
| **Covariates*** | **Unvaccinated (%) 169,568 (81.8)** | **3-doses vaccine (%) 37,841 (18.2)** | **Standardized differences** | **Unvaccinated (%) 31,978 (50.0)** | **3-doses vaccine (%) 31,978 (50.0)** | **Standardized differences** |
| Age group (years) | | | | | | |
| 12 - 17 | 1,526 (14,4) | 1,610 (80,1) | reference | 1,465 (78,5) | 1,467 (78,6) | reference |
| 5 – 11 | 2,786 (26,2) | 131 (6,5) | -0.005907 | 136 (7,3) | 131 (7,0) | 0.001138 |
| 0 - 4 | 6,319 (59,4) | 268 (13,3) | -0.019053 | 265 (14,2) | 268 (14,4) | 1.05e-15 |
| Sex | | | | | | |
| Female | 4,926 (46,3) | 1,124 (55,9) | reference | 1,009 (54,1) | 1,015 (54,4) | reference |
| Male | 5,704 (53,7) | 885 (44,1) | -0.055224 | 857 (45,9) | 851 (45,6) | 0.000063 |
| Region | | | | | | |
| Southeast | 5,394 (50,7) | 1,289 (64,2) | reference | 1,038 (55,6) | 1,182 (63,3) | reference |
| South | 1,537 (14,5) | 243 (12,1) | -0.180872 | 243 (13,0) | 237 (12,7) | -0.000538 |
| Central-West | 1,441 (13,6) | 157 (7,8) | -0.131888 | 262 (14,0) | 155 (8,3) | 0.000678 |
| Northeast | 1,522 (14,3) | 252 (12,5) | 0.018506 | 224 (12,0) | 224 (12,0) | -0.000273 |
| North | 737 (6,9) | 68 (3,4) | -0.095381 | 99 (5,3) | 68 (3,6) | 0.000905 |
| Ethnicity | | | | | | |
| White | 4,086 (48,0) | 901 (57,6) | reference | 681 (47,7) | 840 (57,6) | reference |
| Brown | 3,993 (46,9) | 517 (33,0) | -0.119614 | 634 (44,4) | 483 (33,1) | -0.000277 |
| Black | 287 (3,4) | 70 (4,50 | 0.025933 | 57 (4,0) | 63 (4,3) | -0.000779 |
| Asian | 124 (1,5) | 77 (4,9) | 0.090667 | 56 (3,9) | 72 (4,9) | 0.000865 |
| Indigenous | 15 (0,2) | 0 (0,0) | 0.009133 | 1 (0,1) | 0 (0,0) | 0.010611 |

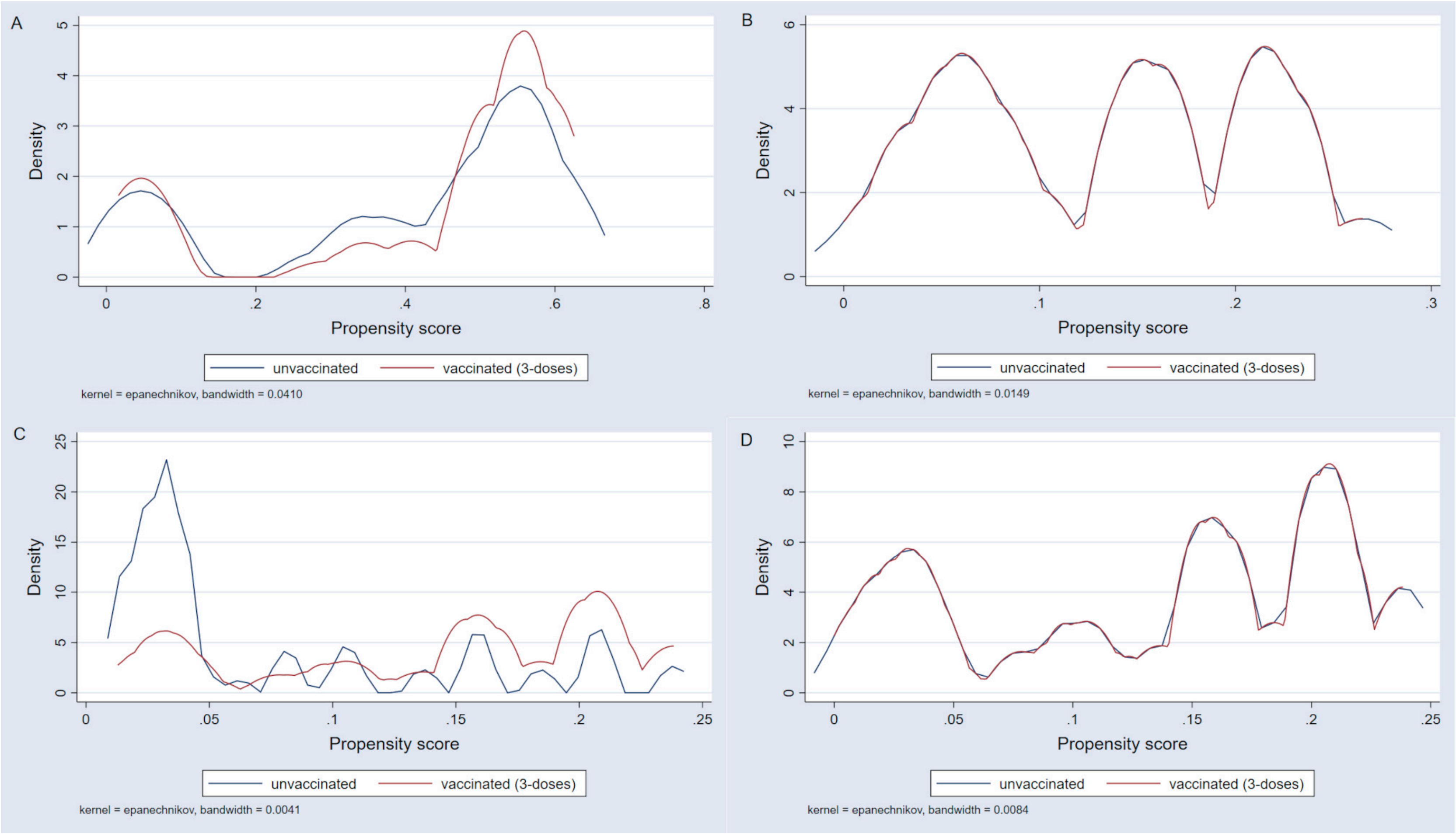


**Figure S2.** Distribution of propensity scores for unvaccinated and vaccinated individuals in pandemic era. (A) patients with comorbidities, before matching; (B) patients with comorbidities, after matching; (C) patients without comorbidities, before matching; and (D) patients without comorbidities, after matching.

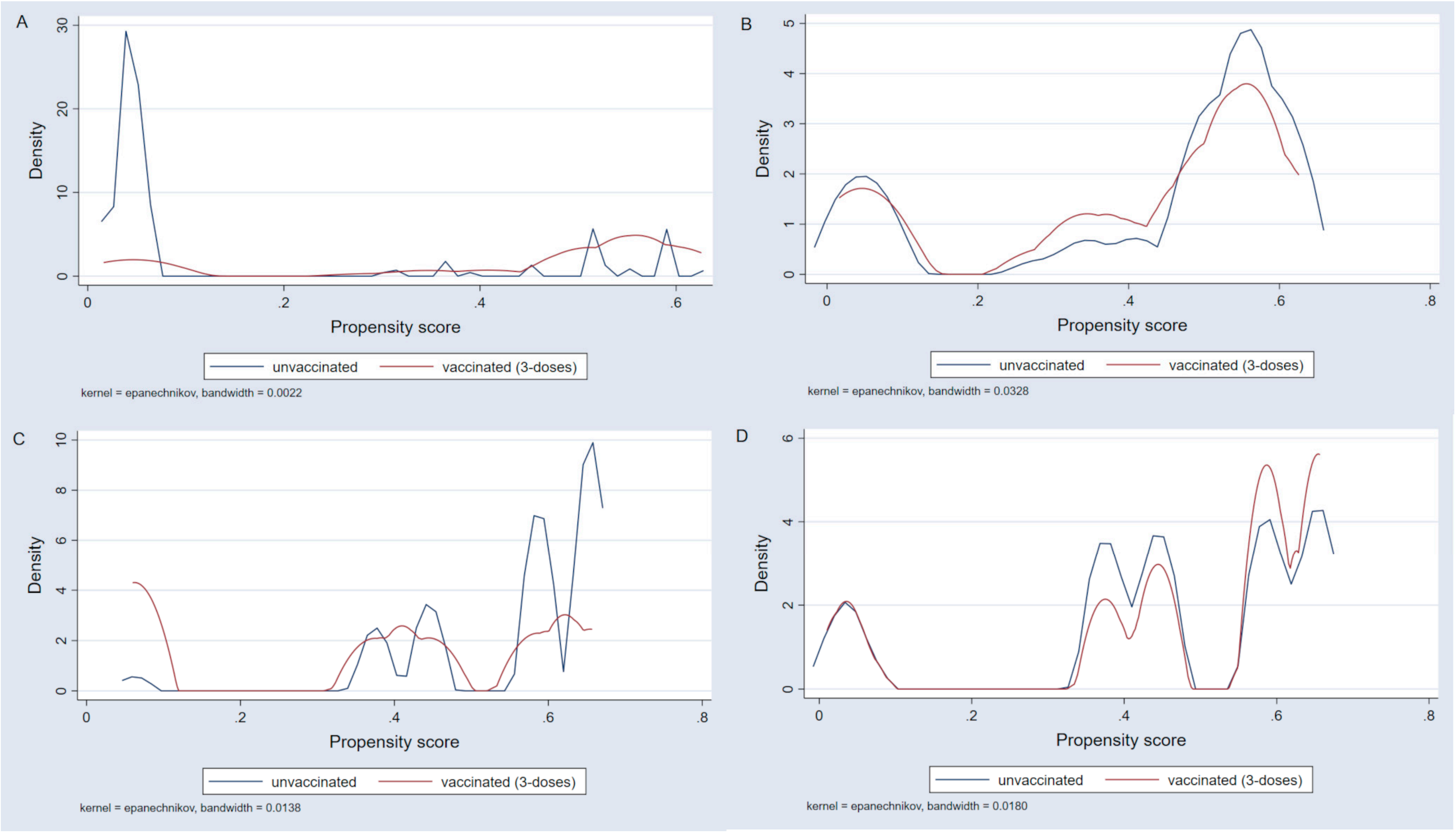


**Figure S3.** Distribution of propensity scores for unvaccinated and vaccinated individuals in post-pandemic era. (A) patients with comorbidities, before matching; (B) patients with comorbidities, after matching; (C) patients without comorbidities, before matching; and (D) patients without comorbidities, after matching.
